# Activation of the mTOR signaling pathway is required for asthma onset

**DOI:** 10.1038/s41598-017-04826-y

**Published:** 2017-07-03

**Authors:** Yanli Zhang, Ying Jing, Junying Qiao, Bin Luan, Xiufang Wang, Li Wang, Zhe Song

**Affiliations:** 1 Department of Pediatrics, the Third Affiliated Hospital of Zhengzhou University, Zhengzhou, Henan 450052 China; 2 School of Medicine for Basic Research, Zhengzhou University, Zhengzhou, Henan 450001 China

## Abstract

The mTOR pathway has been implicated in immune functions; however, its role in asthma is not well understood. We found that patients experiencing an asthma attack, when compared with patients in asthma remission, showed significantly elevated serum mTOR pathway activation, increased Th17 cells and IL-4, and decreased Treg cells and IFN-γ. In patients experiencing asthma, mTOR activation was positively correlated with the loss of Th17/Treg and Th1/Th2 balance. The role of mTOR in asthma was further confirmed using an ovalbumin-induced asthmatic mouse model. The mTOR pathway was activated in asthmatic mice, demonstrated by elevated levels of p-PI3K, p-Akt, p-mTOR, and p-p70S6k, and this activation was significantly reduced by treatment with budenoside or mTOR pathway inhibitors. Moreover, mTOR pathway inhibitor treatment reduced asthmatic markers and reversed the Th17/Treg and Th1/Th2 imbalances in asthmatic mice. Finally, different mTOR pathway inhibitor treatments have different inhibitory effects on signaling molecules in asthmatic mice. In summary, mTOR is activated during asthma onset and suppressed during asthma remission, and inhibiting the mTOR pathway in asthmatic mice alleviates asthmatic markers and restores the balances of Th17/Treg and Th1/Th2 cytokines. These data strongly suggest a critical requirement for mTOR pathway activation in asthma onset, suggesting potential targets for asthma treatments.

## Introduction

Asthma is a common chronic inflammatory disease of the airways and a severe health risk for children^1^. Seventy to eighty percent of asthma onset happens when children are 5 years old or younger, which, if not diagnosed or treated appropriately, will lead to airway remodeling and more severe health consequences. To date, despite the progress on asthma diagnosis and treatment, there are still limited treatment options^2, 3^. Therefore, it is critical to understand the molecular mechanisms of asthma onset and develop targeted therapy accordingly.

Loss of Th1/Th2 balance is believed to be a critical factor in asthma pathogenesis^4^. A Th1/Th2 imbalance can be triggered by changes in the levels of IFN-γ and IL-4 secreted by Th1 and Th2 cells, respectively^4^. Recent studies suggest that the loss of balance between Th17 and regulatory T cells (Treg) also occurs in asthma pathogenesis^5^. IL-17, secreted by activated Th17, regulates pulmonary inflammation in airway smooth muscle cells and fibroblasts^6^. Treg, differentiated from CD4+ T cells, mediate immune suppression by secreting TGF-β and IL-10. Elevated levels of Th17 cells have been shown in children with asthma, and the number of Th17 cells positively correlates with pulmonary function damage^7^. Further, the levels of IL-17, TGF-β, and IL-10 during asthma onset are good surrogates for the numbers of Th17 and Treg cells^8^.

mTOR, the mechanistic target of rapamycin (formerly the mammalian target of rapamycin), is a serine/threonine kinase that is evolutionarily conserved^9^. It is a central regulator of cell metabolism, growth, proliferation, and survival. In mammals, PI3K is activated by certain stimuli or inflammation. Activated PI3K (p-PI3K) phosphorylates Akt, which then activates mTOR and its downstream effector ribosomal protein S6 kinase 1 (S6K1). Phosphorylated S6K1 (p-p70S6k) promotes protein translation and cell growth, with 100-fold greater efficiency in initiating protein translation than its inactive form^10^. Aberrant mTOR signaling is involved in many diseases, including cancer, cardiovascular disease, and diabetes^9, 11^. In atherosclerosis, mTOR activates macrophage proliferation and promotes endothelial cell migration and foam cell formation^12^. Inhibiting mTOR signaling pathway has been shown to lead to suppression of macrophage autophagy and atherosclerosis^13^. mTOR also regulates lymphocyte cellular immunity by stimulating cytokine release from inflammatory cells^14^. In addition, systemic lupus erythematous was suppressed when patients were treated with the mTOR-inhibitor rapamycin^15^.

Asthma, like atherosclerosis and systemic lupus erythematosus, is a disease with dysregulation of the immune system^16–18^. Thus, we hypothesize that mTOR signaling is also playing a role in asthma disease onset. PI3K/mTOR signaling is reported to be very important for the growth and proliferation of airway smooth muscles^19^, and blocking mTOR with rapamycin suppresses asthmatic airway remodeling^20, 21^. Further, the mTOR pathway has been reported to regulate the differentiation and activation of CD4+ T cell subsets, and treatment with rapamycin leads to T cell anergy^22^. Th2 and Th17 cells are differentiated from T cells, and this differentiation was blocked in mTOR knockout mice^23^. Therefore, we believe that the mTOR signaling pathway is tightly associated with the loss of balance between Th1 and Th2 cytokines and between Th17 and Treg cells in immune diseases. This study focused on the role of mTOR in asthma, whether asthma onset could be suppressed by selectively inhibiting mTOR signaling, and a comparison of the effects of inhibitors targeting different molecules in the pathway.

Inhibitors for the mTOR signaling pathway include: (1) mTOR inhibitors (rapamycin and analogs), (2) PI3K inhibitor (LY294002), (3) Akt inhibitor (triciribine), and (4) PI3K/mTOR dual inhibitor (NVP-BEZ235). Among these, the most well-studied inhibitor is rapamycin, which was first used as an antibiotic and later as an immunosuppressant. The antitumor activity of rapamycin is evidenced by increased apoptosis induced by rapamycin in many types of cancer cells^24^. In our study, we used the mTOR inhibitor rapamycin, the PI3K inhibitor LY294002, and the Akt inhibitor triciribine. By comparing the effects of these inhibitors, we can identify promising asthma treatment targets.

## Results

**Figure 1:**
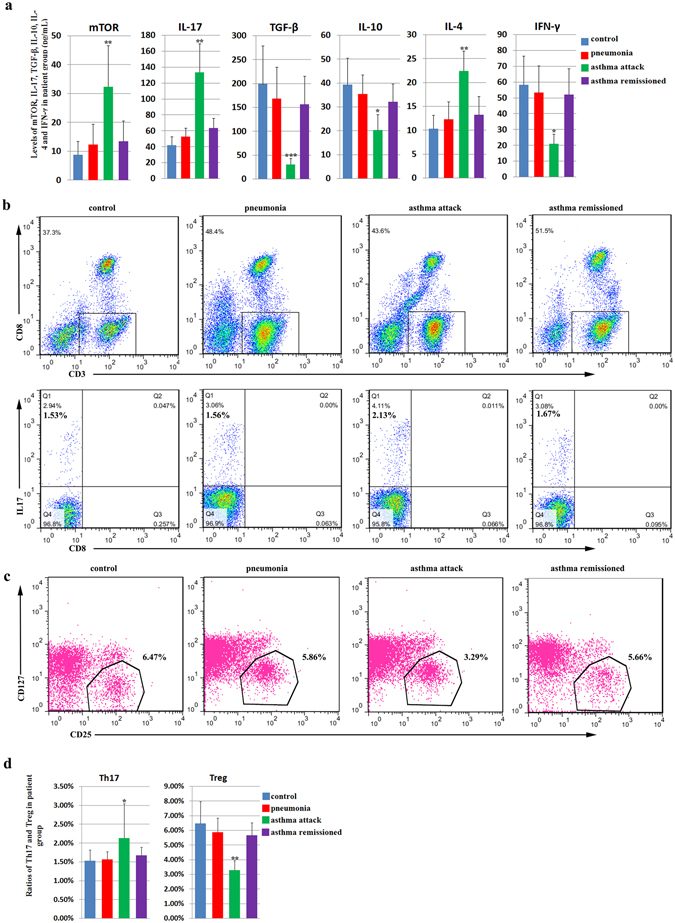
Levels of mTOR, Th17 cells, Treg cells, and asthma-related cytokines in the serum of patients with asthma. The patients were categorized into four groups: the asthma attack group, asthma remission group, community-acquired pneumonia (pneumonia) group, and normal healthy group. The asthma group consisted of children with asthma who visited a doctor or were hospitalized from January 2014 to June 2014. The remission group comprised children who had experienced an asthma attack but were then treated with budenoside and were in asthma remission. The pneumonia group comprised children who were diagnosed with pneumonia in the same hospital in the same period. The healthy control group comprised normal healthy children who had a physical examination in the hospital in the same period. (**a**) The level of serum mTOR (pg/mL), IL-17 (pg/mL), TGF-β (pg/mL), IL-10 (pg/mL), IL-4 (pg/mL), and IFN-γ (pg/mL) detected by ELISA in the control group, the pneumonia group, the asthma attack group, and the asthma remission group. The levels of mTOR, IL-17, and IL-4 in the serum of patients in the asthma attack group were significantly higher than that in the pneumonia and control groups, whereas these elevations were not observed in patients in asthma. Similarly, the levels of TGF-β, IL-10, and IFN-γ in the serum of patients in the asthma attack group were significantly lower than that in the pneumonia and control groups, whereas reduction of these cytokines was not observed in patients in asthma. (**b**) Flow cytometric analysis of Th17 cells (CD3^+^CD8^−^IL-17^+^) in peripheral blood mononuclear cells (PBMC) from patients in the control group, the pneumonia group, the asthma attack, and the asthma remission group. The data was presented as the percentage of all live cells. (**c**) Flow cytometric analysis of Treg cells (CD4^+^CD25^high+^CD127^low^) in PBMC from patients in the control group, the pneumonia group, the asthma attack, and the asthma remission group. (**d**) Levels of Th17 cells (CD3^+^CD8^−^IL-17^+^) and Treg cells (CD4^+^CD25^high+^CD127^low^) detected by flow cytometry in PBMC from patients in the control group, the pneumonia group, the asthma attack group, and the asthma remission group. Th17 and Treg cell percentages in total live cells were presented. The level of Th17 was significantly higher in asthma attack patients than the rest three groups, whereas the level of Treg was significantly lower in asthma attack patients than the rest three groups. All data was presented as means ± SD.*p < 0.05, **p < 0.01, ***p < 0.001.

To examine the correlation between mTOR signaling and asthma, we first compared the levels of mTOR activation in patients experiencing an asthma attack with those in remission, patients with community-acquired pneumonia, and healthy controls. As a result, the mTOR level, determined by ELISA assays, in the serum of patients in the asthma attack group (32.35 ± 14.29 pg/mL) was significantly higher than that in the pneumonia (12.34 ± 7.10 pg/mL) and control (8.73 ± 4.76 pg/mL) groups, whereas elevation of mTOR was not observed in patients in asthma remission (13.41 ± 7.09 pg/mL, Fig. 1a). This indicates that mTOR in patient serum was positively correlated with asthma disease status.

We then examined the correlation of mTOR activation with the loss of balance in Th1/Th2 and Th17/Treg cells. We first measured the levels of cytokines secreted by Th1, Th2, Th17, and Treg cells by ELISA. IL-17 and IL-4 were significantly higher in the asthma attack group than in the other three groups, whereas TGF-β, IL-10, and IFN-γ were significantly lower (Fig. 1a). There were no statistically significant differences in the levels of these cytokines among the remission, pneumonia, and control groups (Fig. 1a). The loss of Th17/Treg balance was then directly determined by measuring the numbers of Th17 and Treg cells in peripheral blood mononuclear cells (PBMC) using flow cytometry. Results showed that the number of Th17 cells (CD3^+^CD8^−^IL-17^+^) in the asthma attack group (2.13 ± 1.78%) was significantly more than that in the asthma remission group (1.67 ± 0.40%), pneumonia group (1.56 ± 0.42%), and control group (1.53 ± 0.56%), whereas the number of Treg cells (CD4^+^CD25^high+^CD127^low^) in the asthma attack group (3.29 ± 1.23%) was significantly less when compared to that in the three remaining groups (5.66 ± 1.65%, 5.86 ± 1.93%, and 6.47 ± 2.87% for the asthma remission, pneumonia, and control groups, respectively, Fig. 1b–d). There were no statistically significant differences in the number of Th17 or Treg cells between the remission, pneumonia, and control groups (Fig. 1b–d). The changes in Th17 and Treg cells, together with the increase in IL-17 levels and the decrease in TGF-β and IL-10 levels, indicate the loss of Th17/Treg balance. Similarly, changes in IL-4 and IFN-γ levels were consistent with the loss of Th1/Th2 balance, which has been reported previously.

**Figure 2:**
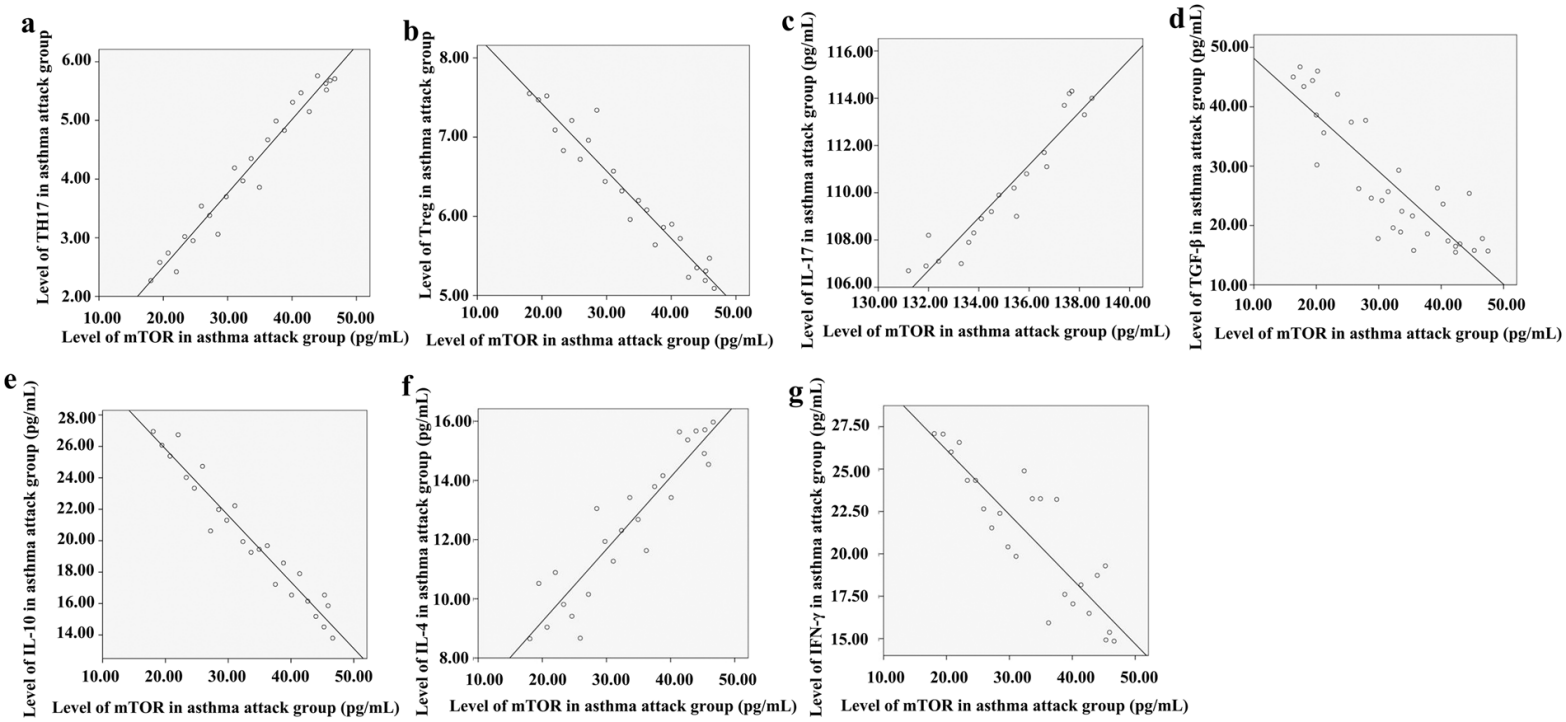
Correlation of serum mTOR levels with the loss of Th17/Treg and Th1/Th2 balances in patients experiencing an asthma attack. The asthma patient group comprised children with asthma who visited a doctor or were hospitalized from January 2014 to June 2014. Serum was collected from the fasting venous blood of these patients, and mTOR as well as the cytokines were measured by ELISA. Peripheral blood mononuclear cells (PBMC) were isolated from the fasting venous blood of these patients, and Th17 and Treg were measured by flow cytometry. (**a**) Positive correlation between the amounts of serum mTOR (pg/mL) determined by ELISA and PBMC Th17 cells (%) determined by flow cytometry in patients experiencing an asthma attack. Correlation analysis performed with Pearson correlation, r = 0.960 and p < 0.001. (**b**) Negative correlation between the amounts of serum mTOR (pg/mL) determined by ELISA and PBMC Treg cells (%) determined by flow cytometry in patients experiencing an asthma attack. Correlation analysis performed with Pearson correlation, r = −0.932 and p < 0.001. (**c**) Positive correlation between the amounts of serum mTOR (pg/mL) and serum IL-17 (pg/mL) determined by ELISA in patients experiencing an asthma attack. Correlation analysis performed with Pearson correlation, r = 0.754 and p < 0.001. (**d**) Negative correlation between the amounts of serum mTOR (pg/mL) and serum TGF-β (pg/mL) determined by ELISA in patients experiencing an asthma attack, r = −0.740 and p < 0.05 by Pearson correlation analysis. (**e**) Negative correlation between the amounts of serum mTOR (pg/mL) and serum IL-10 (pg/mL), determined by ELISA in patients experiencing an asthma attack, r = −0.944 and p < 0.001 by Pearson correlation analysis. (**f**) Positive correlation between the amounts of serum mTOR (pg/mL) and serum IL-4 (pg/mL) determined by ELISA in patients experiencing an asthma attack, r = 0.855 and p < 0.001 by Pearson correlation analysis. (**g**) Negative correlation between the amounts of serum mTOR (pg/mL) and serum IFN-γ (pg/mL) determined by ELISA in patients experiencing an asthma attack, r = −0.762 and p < 0.001 by Pearson correlation analysis.

In patients experiencing an asthma attack, the levels of serum mTOR correlated positively with the number of Th17 cells (Fig. 2a) and negatively with the number of Treg cells (Fig. 2b). Consistent with this, the level of mTOR correlated positively with that of IL-17, but correlated negatively with those of TGF-β and IL-10 (Fig. 2c–e). These data suggest that the level of mTOR and loss of Th17/Treg balance are associated. Similarly, mTOR in patients experiencing asthma was associated with the loss of Th1/Th2 balance, as the level of mTOR in the serum of these patients correlated positively with the level of IL-4 (Fig. 2f) and negatively with the level of IFN-γ (Fig. 2g). There was no correlation between mTOR and any of these cytokines in the remission, pneumonia, or control groups (data not shown). These data suggest a strong association between mTOR activation and the loss of Th17/Treg balance and Th1/Th2 balance in asthma. In the asthma remission phase, mTOR, Th17, Treg cells, and their cytokines were restored to similar levels as those in the controls, indicating that inflammation during asthma was suppressed in the asthma remission phase to a level similar to that of the controls.

### mTOR pathway is activated in asthmatic mouse models

To further define the role of mTOR in asthma onset and to validate our observations in human specimens, we established an asthmatic mouse model by treating mice with ovalbumin (OVA)/aluminum, and alleviated their asthma by pre-treating the mice with budenoside. Three days into OVA stimulation, mice in the asthmatic group displayed restlessness, sneezing, and deep breathing, which stopped about 10 minutes after completing the 30-minute OVA stimulation. Five days into OVA stimulation, mice in the asthma group showed either a significant increase or decrease in physical activity, whereas mice in the control group behaved as they did before the experiment.

**Figure 3:**
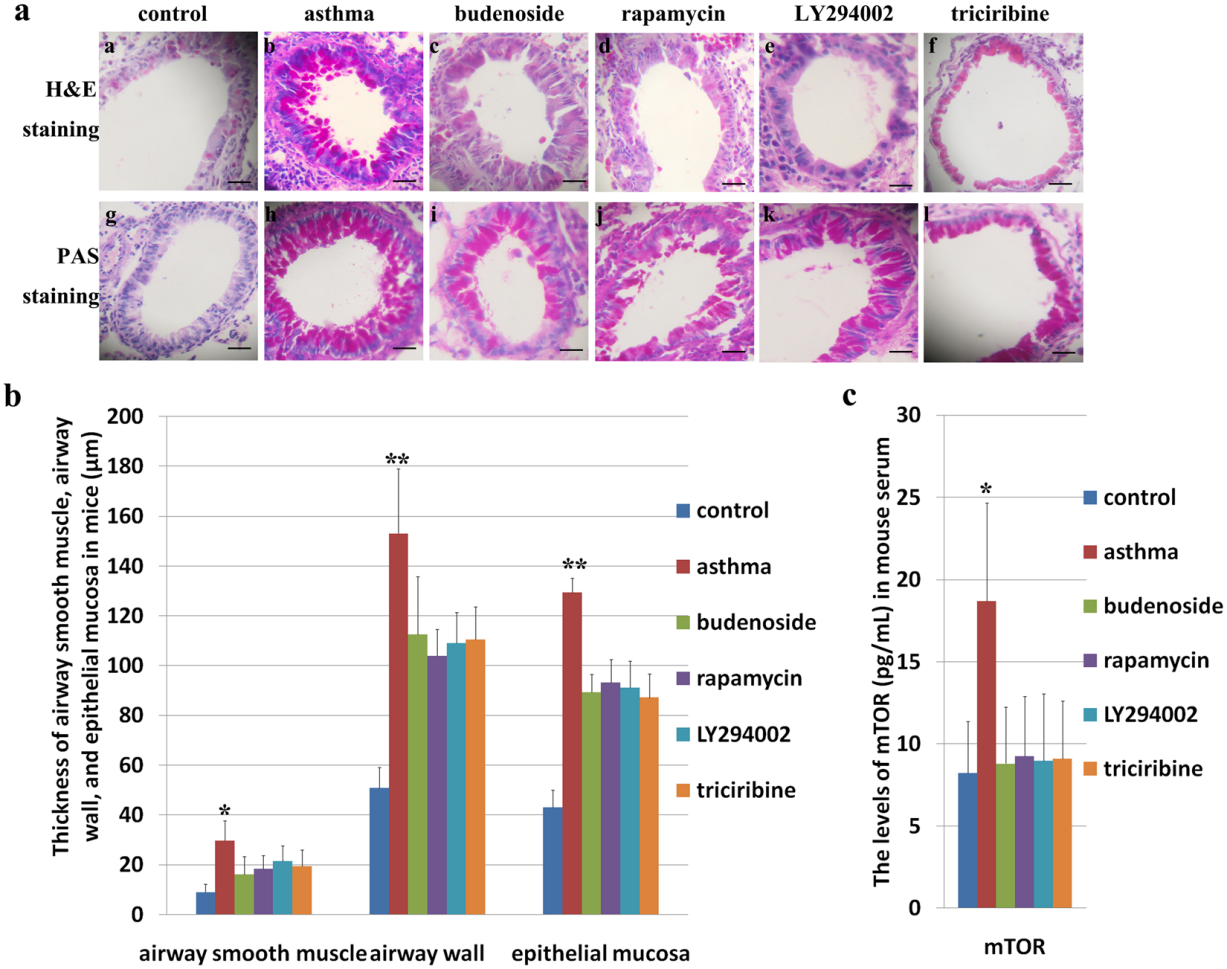
Pathological changes in the lung tissues of asthmatic mice and asthmatic mice treated with budenoside or mTOR pathway inhibitors. Mice were injected intraperitoneally with 0.2 mL ovalbumin (OVA)/aluminum hydroxide on days 1, 8, and 15, and then subjected to 2% OVA inhalation for 30 minutes of stimulation every other day, starting from day 22 for a total of 10 doses, to induce asthma onset. Asthma was established in the treatment groups using the same procedures, except with an additional 30 minutes of inhalation treatment with 1 mg (2 mL) budesonide (**c**) or intraperitoneal injection of rapamycin ((**d**) 3 mg/kg), LY294002 ((**e**) 1 mg/kg), or triciribine ((**f**) 1 mg/kg) before stimulation. (**a**) H&E staining (upper) and PAS staining (lower) of mouse lung tissues from the control group, from the asthma group, and from asthmatic mice treated with budenoside, rapamycin, LY294002, or triciribine. All images were obtained at 200× magnification. The scale bar represents 80 μm. (**b**) Thickness of the airway smooth muscle (μm), airway wall (μm) and epithelial mucosa (μm) determined by H&E staining of tissues from mice in the control group, the asthma group, and the groups treated with budenoside, rapamycin, LY294002, or triciribine. The asthmatic mice demonstrated significantly higher levels of pathological changes than in the control and other treatment groups. (**c**) The levels of mTOR (pg/mL) in mouse sera from the control group, the asthma group, and the groups of asthmatic mice treated with budenoside, rapamycin, LY294002, or triciribine. The asthmatic mice demonstrated significantly higher levels of mTOR than in the control and other treatment groups. All data was presented as the mean ± SD. *p < 0.05, **p < 0.01, ***p < 0.001.

**Figure 4:**
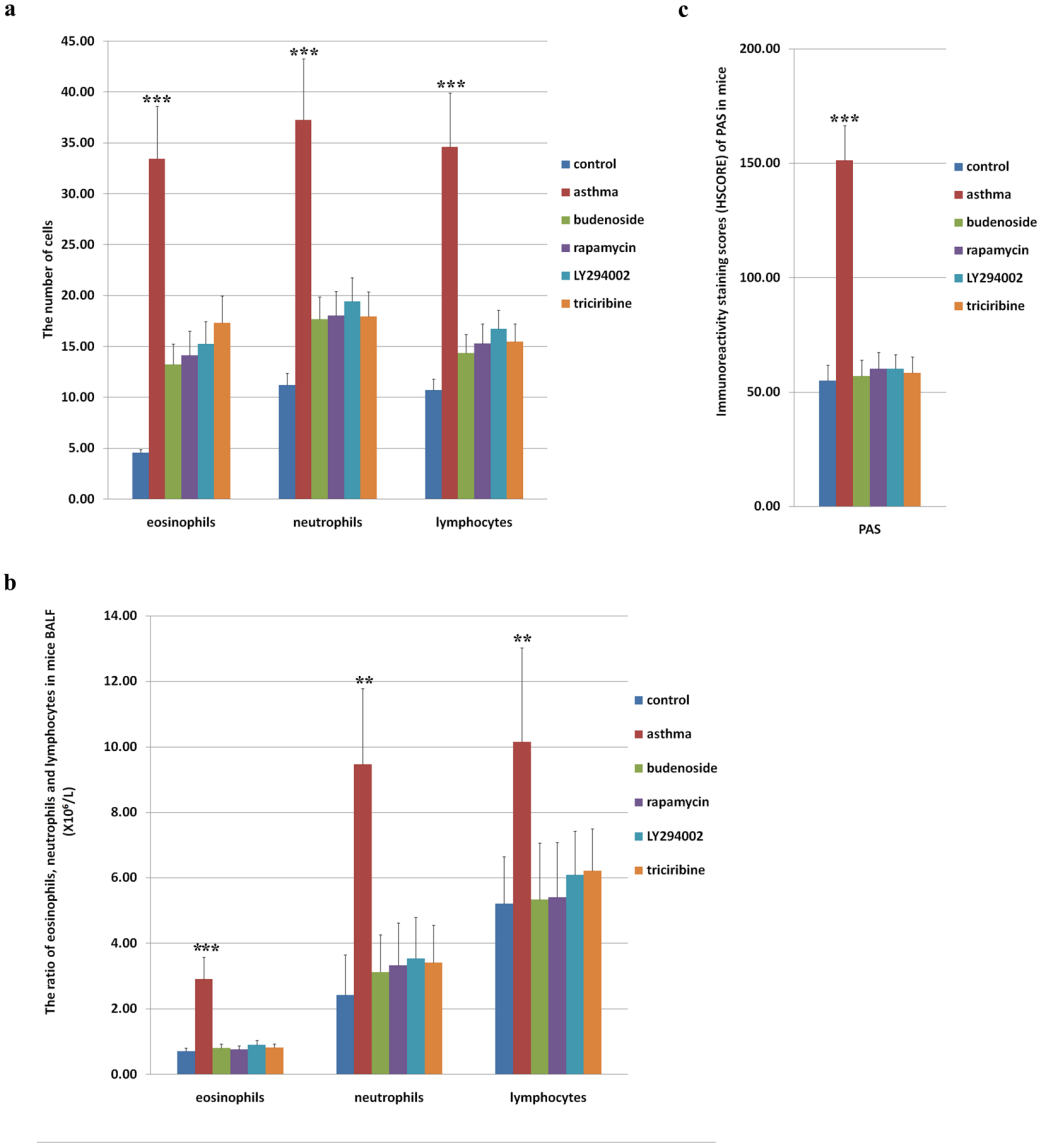
Inflammatory cells in mouse lung tissues and bronchoalveolar lavage fluid (BALF) after treatment with mTOR inhibitors. (**a**) The average number of inflammatory cells, namely eosinophils, neutrophils, and lymphocytes, determined by H&E staining, in the lung tissue from mice in the control group, the OVA-induced asthma group, and the OVA-induced asthmatic group that were treated with budenoside, rapamycin, LY294002, or triciribine. The asthmatic mice demonstrated significantly higher levels of inflammatory cells than in the control, budenoside treatment, and m-TOR pathway inhibitor treatment groups. (**b**) Ratio of inflammatory cells (eosinophils, neutrophils, and lymphocytes) in mouse BALF detected by flow cytometry in mice from the control group and the asthma group, and the asthmatic mice that were treated with budenoside, rapamycin, LY294002, or triciribine. The asthmatic mice demonstrated significantly higher levels of inflammatory cells than in the control, budenoside treatment, and m-TOR pathway inhibitor treatment groups. (**c**) Immunoreactivity staining score (HSCORE) of PAS-positive cells in lung tissue from mice in the control group, the OVA-induced asthma group, and the OVA-induced asthmatic group that were treated with budenoside, rapamycin, LY294002, or triciribine. The asthmatic mice demonstrated significantly higher levels of PAS-positive cells than in the control, budenoside treatment, and m-TOR pathway inhibitor treatment groups. All data was presented as the mean ± SD. *p < 0.05, **p < 0.01, ***p < 0.001.

We first confirmed that the asthmatic mouse model was established by analyzing the pathological changes in normal control mice, asthmatic mice, and budenoside-treated mice. Hematoxylin and eosin (H&E) staining of lung tissues from the asthmatic mice demonstrated submucosal edema, mucous gland hyperplasia, and increased mucous secretion, accompanied by increased mucosal folds, visible epithelial fractures, epithelial cell shedding, mild bronchiole smooth muscle hypertrophy, as well as bronchial wall and basement membrane thickening and irregularities in its shape (Fig. 3a and b). H&E staining of lung tissue from asthmatic mice also showed excessive infiltration of inflammatory cells (including eosinophils, neutrophils, and lymphocytes) into the bronchial submucosa, bronchial, and perivascular spaces (Fig. 4a). Elevated numbers of inflammatory cells in mouse bronchoalveolar lavage fluid (BALF) were also observed in the asthmatic mice when compared to the other groups (Fig. 4b). Similar but milder pathological changes were observed in the lung tissue samples from mice in the budesonide intervention group, whereas none of these pathological changes was observed in the normal control group (Figs 3 and 4). Consistent with the H&E staining results, periodic acid-Schiff (PAS) staining of mouse lung tissues also showed more PAS-positive cells in the asthmatic group than in the control and budenoside treatment groups (Fig. 4c). Consistent with the observation of elevated serum mTOR in patients experiencing asthma, the asthmatic mice showed significantly enhanced serum mTOR levels (Fig. 3c). These results confirmed the activation of mTOR during asthma onset, demonstrated the establishment of the asthmatic mouse model, and indicated that asthma treatment with budenoside was effective.

**Figure 5:**
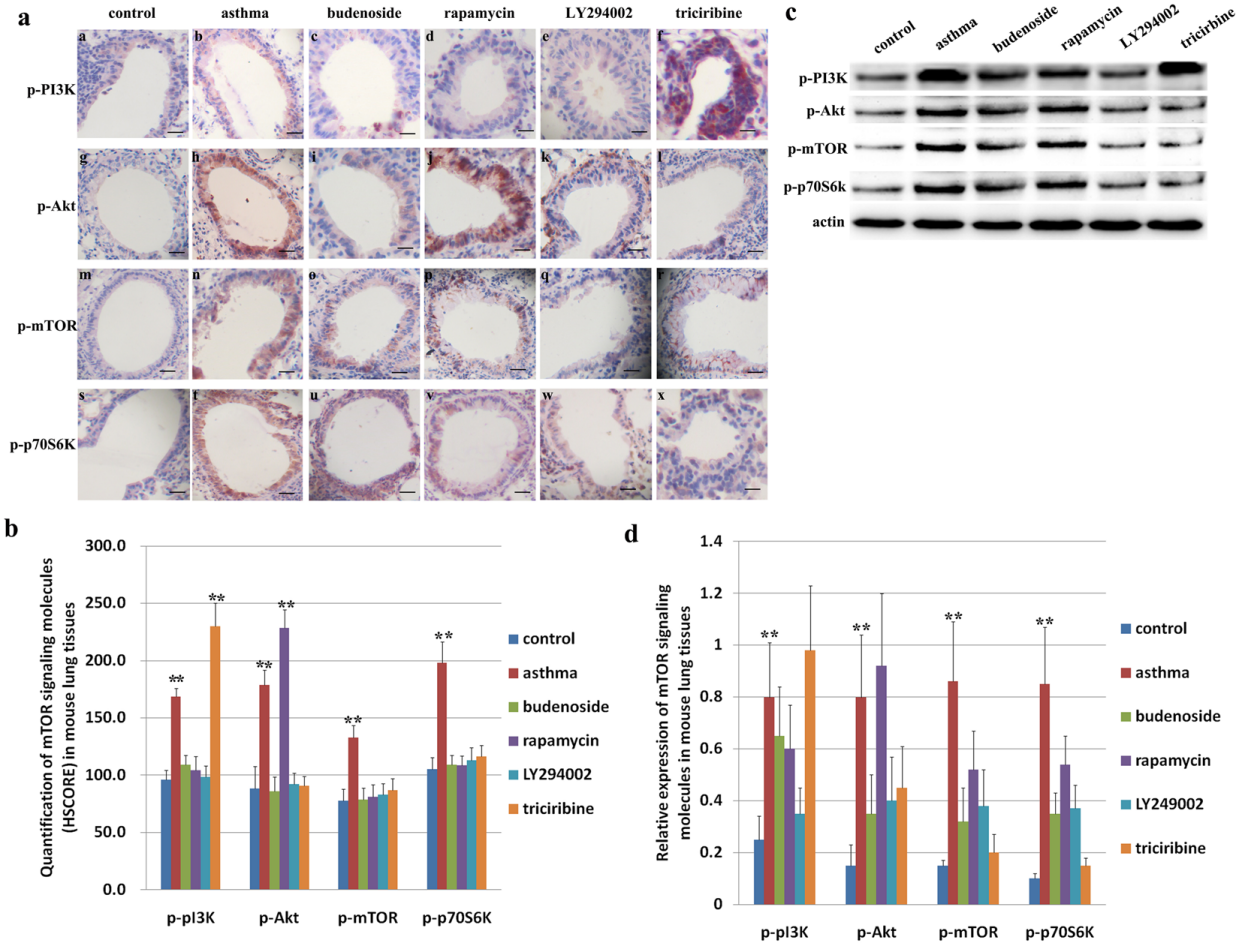
Activation of mTOR signaling molecules in the lung tissues of asthmatic mice and asthmatic mice treated with budenoside or mTOR pathway inhibitors. (**a**) IHC staining of mTOR signaling molecules of tissues from mice in the control group and the OVA-induced asthma group, and the OVA-induced asthmatic mice that were treated with budenoside, rapamycin, LY294002, or triciribine. All images were obtained at 200× magnification. The scale bar represents 80 μm. (**b**) Quantification of IHC staining (HSCORE) of mTOR signaling molecules from mice in the control group, the OVA-induced asthma group, and the OVA-induced asthmatic mice treated with budenoside, rapamycin, LY294002, or triciribine. The asthmatic mice demonstrated significantly higher levels of p-mTOR, and p-p70S6k than in the control, budenoside treatment, and m-TOR pathway inhibitor treatment groups. No statistical significance was observed among those groups. The level of p-PI3K in the asthmatic group and the triciribine-treated group were significantly higher than the rest of the groups. The level of p-Akt in the asthmatic group and the rapamycin-treated group were significantly higher than the rest of the groups. (**c**) Activation of mTOR signaling molecules determined by western blot in lung tissues from mice in the control group, the OVA-induced asthma group, and the OVA-induced asthmatic mice treated with budenoside, rapamycin, LY294002, or triciribine. (**d**) Quantification of mTOR signaling molecules by western blot normalized against the expression of β-actin. The asthmatic mice demonstrated significantly higher levels of p-mTOR, and p-p70S6k than in the control, budenoside treatment, and m-TOR pathway inhibitor treatment groups. No statistical significance was observed among those groups. The level of p-PI3K in the asthmatic group and the triciribine-treated group were significantly higher than the rest of the groups. The level of p-Akt in the asthmatic group and the rapamycin-treated group were significantly higher than the rest of the groups. All data was presented as the mean ± SD. *p < 0.05, **p < 0.01, ***p < 0.001.

We then used immunohistochemical (IHC) staining to examine activation of the mTOR pathway during asthma onset in the normal control, asthmatic, and budenoside-treated mice. Staining of mTOR signaling molecules was mainly concentrated around alveolar epithelial cells, as well as macrophages and neutrophils in the bronchioles (Fig. 5a). Consistent with the activation of mTOR in patients experiencing an asthma attack, we observed significantly higher levels of p-PI3K, p-Akt, p-mTOR, and p-p70S6k in asthmatic mice than in the control and budenoside treatment groups (Fig. 5a and b). No statistically significant difference in the activation of mTOR signaling molecules was observed between the control and budenoside-treated group (Fig. 5a and b). The elevated levels of mTOR in asthmatic mouse models, together with the observation of mTOR activation in asthma patients, suggests that the mTOR pathway is turned on during asthma disease onset.

### Treatment with mTOR pathway inhibitors alleviated asthmatic markers in mice

To determine whether activation of mTOR is required for asthma onset, we examined whether mTOR pathway inhibitors could alleviate asthmatic markers in mice. In the intervention, OVA/aluminum-induced asthmatic mice were pre-treated with budenoside or mTOR pathway inhibitors LY294002, triciribine, or rapamycin. H&E staining of lung tissue from the asthmatic mice treated with mTOR inhibitors showed that asthmatic markers were less pronounced than those in untreated asthmatic mice. Pathological changes, including submucosal edema, mucous gland hyperplasia, increased mucous secretion, and increased mucosal folds were observed in mice treated with mTOR inhibitors, but were much milder than in mice that were untreated (Fig. 3a and b). Similarly, mTOR inhibitor treatment decreased epithelial fractures, epithelial cell shedding, bronchiole smooth muscle hypertrophy, as well as bronchial wall and basement membrane thickening and irregularities in its shape (Fig. 3a and b). Mice in the asthma group showed severe airway remodeling, which was improved with budenoside or mTOR pathway inhibitor treatment (Fig. 3a). Mice in the asthma group displayed the highest smooth muscles hypertrophy, thickest airway wall, and the most severe basement membrane thickening, which were all significantly improved with mTOR inhibitor treatment (Fig. 3a and b). Compared to the severe pathological changes in asthmatic mice, mice treated with mTOR inhibitors showed less infiltration of inflammatory cells into the bronchial submucosa, bronchial, and perivascular spaces (Fig. 4a and b). Consistent with the pathological changes observed with H&E staining, PAS staining of mouse lung tissues indicated less PAS-positive cells in the budenoside-treated and mTOR inhibitor-treated mice than in the untreated asthmatic mice (Figs 3a and 4a). This significant improvement in asthmatic markers by mTOR inhibitor treatment suggests that mTOR pathway activation is critical for asthma pathogenesis.

To confirm inhibition of the mTOR pathway by the inhibitors and to further study the inhibitory effect of inhibitors on individual target molecules in the mTOR pathway, we examined p-PI3K, p-Akt, p-mTOR, and p-p70S6k by IHC staining of lung tissue from mice in all groups. Activation of these molecules was mainly seen in pulmonary alveolar epithelial cells, bronchial epithelial cells, macrophages, and neutrophils (Fig. 5a). Mice in the asthma group showed significantly higher levels of mTOR activation than those in the control group, and this elevated mTOR activation was significantly reduced with budenoside, LY294002, triciribine, or rapamycin treatment. There were no statistically significant differences in IHC staining between the intervention groups (Fig. 5a and b). Similarly, p-p70S6k showed the highest expression levels in the asthma group. These levels were significantly higher than those in the control group or the groups of mice treated with budenoside, LY294002, triciribine, or rapamycin. There were no statistically significant differences between the intervention groups (Fig. 5a and b). The level of p-PI3K in the asthma group was significantly higher than that in the control group and the budenoside treatment group. PI3K inhibitor LY294002 treatment and mTOR inhibitor rapamycin treatment significantly reduced the level of p-PI3K to levels comparable to those in the control and budenoside treatment group. Interestingly, the mice treated with the Akt inhibitor triciribine showed significantly higher levels of p-PI3K than mice in the other groups (Fig. 5a and b). Similar changes were observed with p-Akt levels. The level of p-Akt in the asthma group was significantly higher than that in the control and budenoside treatment groups. The PI3K inhibitor LY294002 treatment group and Akt inhibitor triciribine treatment group showed significant reductions in p-Akt levels, to levels comparable to those of the control and budenoside treatment groups. The mTOR inhibitor rapamycin treatment group showed the highest level of p-Akt among all the groups (Fig. 5a and b). The activation status of these molecules was also confirmed by western blot with proteins extracted from mouse lung tissues (Fig. 5c and d). These results confirmed the inhibition of mTOR signaling molecules with the different inhibitors. The activation of mTOR in asthmatic mice and reduced activation of mTOR in the budenoside treatment group suggests that the mTOR pathway was activated during asthma onset and suppressed when asthma was alleviated. More importantly, the reduced asthmatic markers in response to mTOR inhibitor treatments suggest that activation of the mTOR pathway is required for asthma disease onset.

### mTOR pathway inhibitor treatment reversed the loss of Th1/Th2 and Th17/Treg balances in asthma attack

After confirming activation of the mTOR pathway during asthma onset and demonstrating the requirement of mTOR for asthma disease onset, we then further analyzed whether mTOR pathway suppression can rescue the loss of balance between Th17 and Treg and between Th1 and Th2 cytokines in asthmatic mice during disease onset. The levels of serum mTOR and cytokines in mice were measured by ELISA after asthma induction with or without budenoside or mTOR inhibitor treatment. As a result, mTOR in mouse serum was elevated in the asthmatic mice compared to that in the controls, and this increase was reversed by treatment with budenoside or mTOR pathway inhibitors. There were no significant differences between mTOR levels in the intervention groups (Fig. 3c).

**Figure 6:**
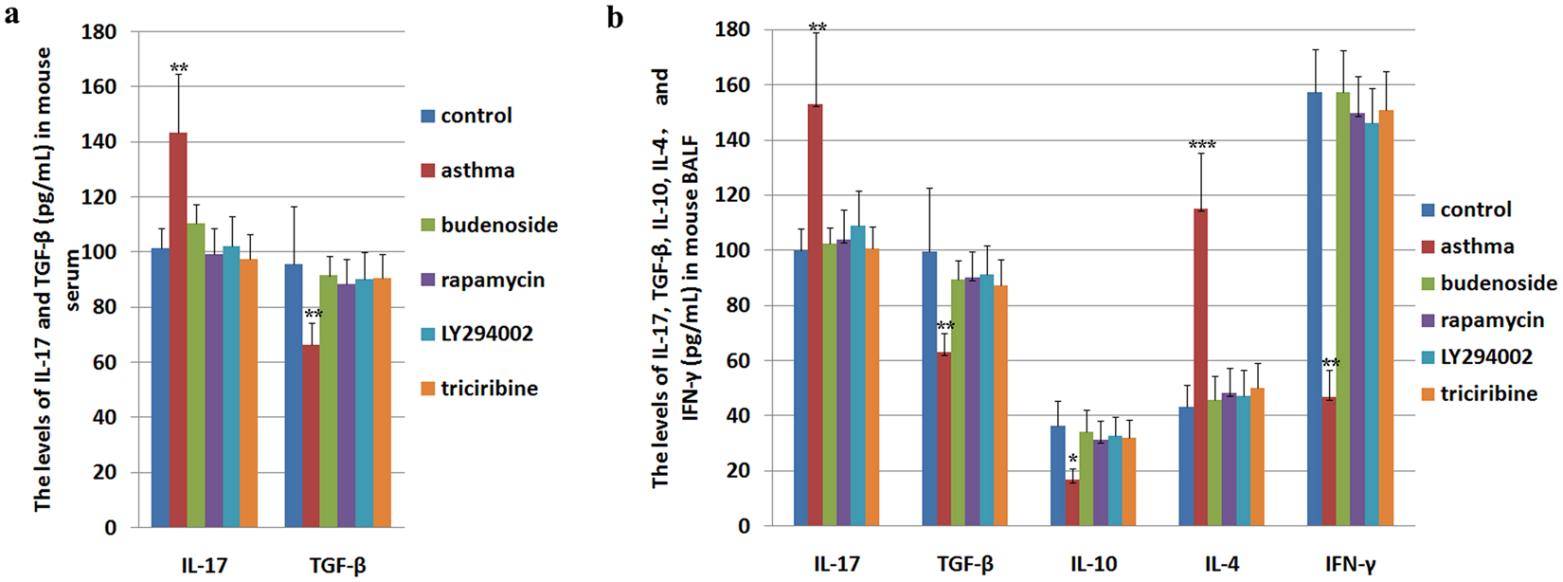
Levels of asthma-related cytokines in mouse serum and bronchoalveolar lavage fluid (BALF) determined by ELISA. (**a**) The levels of IL-17 (pg/mL) and TGF-β (pg/mL) in sera from mice in the control group and the OVA-induced asthma group, and the OVA-induced asthmatic mice treated with budenoside, rapamycin, LY294002, or triciribine. The asthmatic mice demonstrated significantly higher levels of IL-17 and lower level of TGF-β than in the control, budenoside treatment, and m-TOR pathway inhibitor treatment groups. No statistical significance was observed among those groups. (**b**) The levels of IL-17 (pg/mL), TGF-β (pg/mL), IL-10 (pg/mL), IL-4 (pg/mL), and IFN-γ (pg/mL) in BALF from mice in the control group and the OVA-induced asthma group, and the OVA-induced asthmatic mice treated with budenoside, rapamycin, LY294002, or triciribine. The asthmatic mice demonstrated significantly higher levels of IL-17 and IL-4, and lower level of TGF-β, IL-10 and IFN-γ than in the control, budenoside treatment, and m-TOR pathway inhibitor treatment groups. No statistical significance was observed among those groups. All data was presented as the mean ± SD. *p < 0.05, **p < 0.01, ***p < 0.001.

The asthmatic mice showed higher serum IL-17 levels than those in the controls, and this increase was reversed by budenoside or mTOR pathway inhibitor treatment. There were no significant differences between IL-17 levels in the intervention groups (Fig. 6a). BALF IL-17 showed a consistent increase in mice in the asthmatic group and a reduction with budenoside or mTOR pathway inhibitor treatments (Fig. 6b). In contrast, a reduction in serum TGF-β was observed in the asthma group when compared to that in the controls, but was not observed in mice in the asthma group treated with budenoside or the mTOR inhibitor. There were no statistically significant differences between TGF-β levels in the intervention groups (Fig. 6a). BALF TGF-β levels were consistently different from serum TGF-β levels (Fig. 6b). The change in the IL-17/TGF-β ratio in the asthma group was consistent with the Th17/Treg imbalance during asthma onset. Treatment with mTOR inhibitors restored the IL-17/TGF-β ratio in the asthmatic mice, suggesting that inhibiting mTOR pathway activation reversed the loss of Th17/Treg balance during asthma onset.

Similarly, loss of Th1/Th2 balance was observed in the asthmatic mice, demonstrated by the increased BALF IL-4 and decreased BALF IL-10 and IFN-γ when compared to those in the controls. Budenoside treatment reversed the increase in IL-4 and decrease in IL-10 and IFN-γ. Similarly, mTOR inhibitor treatment during asthma onset restored IL-4, IL-10, and IFN-γ levels to levels similar to those in the control mice (Fig. 6a and b). These data suggested that inhibiting mTOR pathway activation reversed the loss of Th1/Th2 balance during asthma onset. Taken together, mTOR pathway activation positively correlated with the loss of Th1/Th2 balance and the loss of Th17/Treg balance during asthma onset, and inhibiting mTOR with mTOR pathway inhibitors restores these balances.

## Discussion

Asthma is a chronic inflammatory disease. Studies have shown that multiple inflammatory cells participate in airway inflammation and induce asthma^25^. Airway remodeling, a major asthmatic symptom, is the biggest challenge in asthma treatment. Our study demonstrated the requirement for mTOR in asthma disease onset and compared the effects of asthma treatment with rapamycin, LY294002, or triciribine to that of the standard budenoside treatment. We demonstrated that mTOR pathway inhibitor treatment not only effectively reduced asthmatic markers, including airway remodeling, in mice but also suppressed the altered Th17/Treg and Th1/Th2 balances that are associated with asthma onset at the molecular level.

Previous studies have suggested the role of mTOR in asthma: airway inflammation was inhibited by reducing the activation of PKC-δ in the mTOR pathway^26^, and airway remodeling was shown to tightly associated with high levels of mTOR expression in an asthmatic mouse model^27^. Further, reducing the levels of p-Akt and p-mTOR suppressed smooth muscle fibrosis in asthma^28^, and rapamycin suppressed asthma onset by inhibiting mTOR signaling^26^. However, despite studies supporting the involvement of mTOR in asthma, controversy exists over the effects of rapamycin treatment in asthma, especially its ability to suppress airway inflammation and airway hyperreactivity^29^. These discrepancies may be a result of different treatment procedures, subjects, or timing, and resolving these differences will require additional investigations. Because of this controversy, there are currently no clinical studies of asthma treatment with rapamycin. In our study of asthma patients, serum mTOR levels in the asthma attack group were significantly higher than those in the remission, pneumonia, and control groups, and the last three groups showed no significant differences, which suggested that the mTOR signaling pathway was activated during asthma and inhibited when asthma was alleviated.

In our animal study, mice in the asthma group demonstrated lung smooth muscle hypertrophy, as well as thickening in airway walls and the epithelial mucosa. These pathological changes, none of which was observed in the control mice, were alleviated with budenoside treatment, indicating the successful establishment of an asthma disease model. The mTOR signaling pathway was activated in the asthma group, and suppression of mTOR activation led to an improvement in asthmatic phenotypes, demonstrating the requirement of mTOR activation for asthma pathogenesis. There were no statistically significant differences between the treatment groups, indicating that p-PI3K, p-Akt, p-mTOR, and p-p70S6k offer four novel potential targets for the next generation of asthma treatments as alternatives to the current standard of budenoside.

Interestingly, the mTOR signaling pathway displayed different patterns of suppression with the different inhibitors. p-mTOR and p-p70S6k levels, highest in the asthma group, were decreased to similar levels in all treatment groups as a result of mTOR pathway suppression by the inhibitors. PI3K activation was highly induced in the asthma group and reduced in the mTOR inhibitor treatment groups, except for the triciribine treatment group, which showed the highest levels of p-PI3K. A similar phenomenon was seen with Akt activation, which was highest in the rapamycin treatment group and second highest in the asthma group. These results indicate a possible negative feedback loop, demonstrated by the compensatory activation of the molecule upstream of the one targeted by the inhibitor in the mTOR pathway.

It is well established that the loss of balance between Th1 and Th2 plays a critical role in asthma onset^30^. In recent years, it has been recognized that a Th1/Th2 imbalance does not fully explain the etiology of asthma, because restoring the Th1/Th2 balance does not completely control asthmatic symptoms in humans^31^. Some studies have suggested that other CD4+ T cell subsets may play a role in asthma, including Th17 and Treg cells^32^. CD4^+^CD25^+^Foxp3^+^ Treg cells may be involved in the pathogenesis of bronchial asthma and may be the upstream regulatory mechanism for the restoration of Th1/Th2 balance by *Lactobacillus salivarius* ^33^. Inactivated atomized *Mycobacterium phlei* suppressed airway inflammation and partially suppressed airway hyperreactivity via modulating the balance between CD4^+^CD25^+^ Treg and Th17 cells^34^. The ratio of Th17/Treg cells plays an important role in regulating allergies and autoimmune diseases. A high level of Treg cells protects against asthma, as evidenced by the alleviation of asthma airway remodeling after replacing endogenous, deficient Treg cells with exogenous, functional Treg cells^35^.

Lung tissues from asthmatic mice show decreased Treg expression and increased Th17 expression. The increased Th17/Treg ratio leads to an increase in the number of infiltrating neutrophils and eosinophils, which in turn induces asthma. In other words, asthma is induced by an imbalance in Th17/Treg cells^36^. Studies have shown that Treg cells regulate cellular immunity through TGF-β and IL-10^37^. IL-6 and TGF-β are critical to determining which direction the Th17/Treg balance will tip^38^. Our study showed a significant decrease in Treg cells, TGF-β, IL-10, and IFN-γ and a significant increase in Th17 cells, IL-17, and IL-4 in both BALF and sera from asthmatic mice. This confirms a loss of Th17/Treg balance, in addition to the loss of Th1/Th2 balance, with asthma disease onset.

mTOR signaling pathway regulates the differentiation and activation of CD4+ T cells^39^. Progesterone promotes the generation and maintenance of Treg cells by suppressing mTOR, indicating the tight association between mTOR activation and the loss of Th17/Treg balance^40^. Our study shows that mTOR activation positively correlates with IL-17 levels and negatively correlates with TGF-β and IL-10 levels, which is consistent with the results of previous studies on the effect of mTOR on CD4^+^ T cell subpopulation differentiation. These results, together, suggest that the downregulation of TGF-β and IL-10 and the upregulation of IL-17 resulting from mTOR activation may cause a loss of balance between Treg and Th17, which induces asthma onset and progression. Because the mTOR signaling pathway is required for asthma onset and progression, and because inhibiting mTOR activation restores the Th17/Treg balance, mTOR inhibitors such as rapamycin represent novel treatment options for asthma.

In our clinical study, asthma attack patient samples were collected before the patients were treated with steroid budenoside, whereas the asthma remission patients were sampled following remission, either with steroid budenoside and β2-adrenergic receptor agonists Terbutaline Sulphate Solution for Nebulization, or auto-remission without any treatment. It is possible that the difference observed in asthma attack patients and remission patients were due to different treatments, however, there were no statistical significances between patients in the remission group with or without treatment in serum mTOR, Th17, Treg, IL-17, TGF-β, IL-10, IL-4, and IFN-γ (Supplemental Table 1), which indicates that the differences observed between asthma attack patients and asthma remission patients were not attributed to steroid or β2-adrenergic receptor agonists treaments.

Currently, budesonide is the standard treatment for asthma, and there are no clinical trials on treating asthma with rapamycin. Our model shows that mTOR inhibitor treatments showed no statistically significant differences in effects from that of budesonide treatment, and we propose mTOR inhibitors as promising potential treatments for asthma that will be at least comparable to budesonide. The effect of mTOR inhibitors on asthma of different subtypes and stages should be investigated.

## Materials and Methods

### Ethics statement

All methods were carried out in accordance with relevant guidelines and regulations. All experiment protocols were approved by a named institutional and/or licensing committee. Specifically, for the patient study, all experiment protocols were reviewed and approved by the Medical Ethics Association of the Third Affiliated Hospital of Zhengzhou University, and written informed consents were received from the parents of all children involved; for animal studies, all protocols were reviewed and approved by the Animal Ethics Committees of the Third Affiliated Hospital of Zhengzhou University under University Animal Research Guideline 1996–21. Animals were housed and treated under the approved protocols, and all efforts were made to minimize animal suffering.

### Clinical study

The patients were categorized into four groups: an asthma attack group, asthma remission group, community-acquired pneumonia group, and normal healthy group. The asthma group consisted of 34 children (17 males, 17 females), aged 4.2 to 10.8 years (mean = 6.5 ± 2.1 years), with asthma who had visited a doctor or were hospitalized in the Third Affiliated Hospital of Zhengzhou University from January 2014 to June 2014. Patients were sampled within 24 hours of first experiencing increased symptoms when they visited the doctor or were hospitalized. Asthma attack patient were treatment with steroid budenoside after sampling. The remission group included 35 children (18 males, 17 females), aged 3.8 to 10.2 years (mean = 6.4 ± 1.8 years), who had experienced an asthma attack and then were in asthma remission either through treatment with the steroid budenoside and β2-adrenergic receptor agonists (Terbutaline Sulphate Solution for Nebulization, 10 males and 9 females) or self-remission without treatment (8 males and 8 females). Patients were sampled within 24 hours of entering remission. There were no statistical significances between patients in the remission group with or without treatment in serum mTOR, Th17, Treg, IL-17, TGF-β, IL-10, IL-4, and IFN-γ (Supplemental Table 1). All of the patients with asthma were confirmed as cases according to the published diagnostic standard^41^. The pneumonia group comprised 37 children (20 males, 17 females), aged 3.7 to 9.9 years (mean = 6.5 ± 1.9 years), who were diagnosed with pneumonia in the same hospital in the same period as the patients with asthma. All of the patients with pneumonia were confirmed as cases according to the published diagnostic standard^41^. The healthy control group comprised 31 normal healthy children (17 males, 14 females), aged 2.9 to 9.3 years (mean = 5.9 ± 1.4 years), that were randomly chosen from those who had a physical examination in the hospital in the same period as the patients with asthma and pneumonia. There were no statistically significant differences in ages and gender composition among the groups (p > 0.05).

### Animal study

Sixty specific pathogen-free (SPF) grade 6- to 8-week-old female Balb/C mice were provided by the Zhengzhou University Animal Experiment Center (serial number: SCXK[Yu] 2015–0005) and housed in the Experiment Center of the Third Affiliated Hospital of Zhengzhou University with free access to food and water for one week prior to initiation of the experiment. Mean mouse body weights were 20 ± 2 g as determined by a digital scale before the experiment. Mice were divided into six groups: saline control (A), OVA-induced asthmatic mice (B), asthmatic mice treated with budesonide (C), asthmatic mice treated with the mTOR inhibitor rapamycin (D), asthmatic mice treated with the PI3K inhibitor LY294002 (E), and asthmatic mice treated with the Akt inhibitor triciribine (F). The asthma mouse model was established based on a previous study^42^ with modifications. Briefly, each mouse was injected intraperitoneally with 0.2 mL OVA (Sigma)/aluminum hydroxide on days 1, 8, and 15, and then subjected to 2% OVA inhalation for 30 minutes for stimulation every other day, starting from day 22 for a total of 10 doses. Asthma was established in the treatment groups using the same procedure, except with an additional 30 minutes of inhalation treatment with 1 mg (2 mL) budesonide (C) or intraperitoneal injection of rapamycin (D, 3 mg/kg, Sigma), LY294002 (E, 1 mg/kg, Sigma), or triciribine (F, 1 mg/kg, Sigma) before stimulation. Rapamycin, LY294002, and triciribine were dissolved in dimethyl sulfoxide (DMSO) to create a stock solution that was diluted with sterile phosphate-buffered saline (PBS) before use. Negative control mice were treated under the same protocol as asthmatic mice, except that OVA was replaced with saline solution. No mice from any of the six groups died during the experiments.

### Evaluation of OVA-induced murine model of asthma

Mice were divided into an asthma group and control group. There were no statistically significant differences between these two groups in body weights, activity levels, or reaction to stimuli before the experiment. No swellings or ulcers were observed during or after the intraperitoneal injection. Three days into stimulation, mice in the asthmatic group displayed restlessness, sneezing, and deepened breathing, which stopped about 10 minutes after completing the 30 minutes of OVA stimulation. Five days into OVA stimulation, mice in the asthma group demonstrated either hypomania or a significant reduction in activity levels. Mice in the control group behaved as they did before the experiment, exhibiting healthy appetites, agile movements, and glossy fur. Mice that displayed shortened breath, restlessness, cyanosis, salivation, as well as fecal and urine incontinence, after inhalation of the allergen indicated the successful establishment of the asthma mouse model. More severe reactions included hypopnea or respiratory arrhythmia, respiratory failure, and lethargic. All mice were evaluated by H&E, IHC, and PAS staining for asthmatic markers such as inflammatory cells, thickening of airway smooth muscles, airway walls, and epithelial mucosa.

### BALF cell collection

Mice were anesthetized and stabilized on a wooden board 24 hours after the last stimulation, and their chests were opened for the following procedures. The distal trachea and left main bronchus were ligated, and then each mouse was tracheally intubated with a modified 22 G catheter for a 0.5-mL cold PBS lavage performed three times. BALF was collected with a recycle rate of >85%. Supernatants were collected after 10 minutes of centrifugation at 4 °C and 1500 rpm, and were stored at −20 °C for use in experiments.

### Isolation of sera from humans and mice

Fasting venous blood was collected from patients with asthma and from controls, allowed to clot at room temperature, and centrifuged for 10 minutes at 2000 rpm. Serum was collected from the top layer in the tube, and aliquoted for use in experiments. Mouse blood was collected by sterile retro-orbital bleeding and processed similarly.

### Enzyme-linked immunosorbent assay (ELISA)

ELISA was performed on clinical serum samples, mouse sera, and BALF according to the manufacturer’s instructions. ELISA kits used were the following: Human mTOR ELISA Detection Kit, Human IL-17 ELISA Detection Kit, Human IL-10 ELISA Detection Kit, Human IL-4 ELISA Detection Kit, Human IFN-γ ELISA Detection Kit, Human TGF-β ELISA Detection Kit, Mouse mTOR ELISA Detection Kit, Mouse IL-17 ELISA Detection Kit, Mouse TGF-β ELISA Detection Kit, Mouse IL-10 ELISA Detection Kit, Mouse IL-4 ELISA Detection Kit, and Mouse IFN-γ ELISA Detection Kit, all from R&D Systems.

### Flow cytometry

PBMCs were suspended at a concentration of 1 × 10^6^/mL in RPMI 1640 medium (Gibco) supplemented with 10% fetal bovine serum. To activate cells, PMA + Ion (25 ng/mL + 1 mg/mL) and protein transport inhibitor BFA (1 mg/mL) (both from Sigma) were added, and cells were incubated at 37 °C for 5 hours with 5% CO_2_. The activated cells were stained with FITC-labeled anti-human CD3 and PerCP-labeled anti-human CD8 and then stored in the dark at 4 °C for 20 min. After being washed with PBS, the cells were fixed with 100 mL fixation solution for 15 minutes and then washed again. Permeabilization working solution was added, mixed for 15 minutes, and then washed with PBS. Intracellular staining was performed with the addition of APC-labeled anti-human IL-17, and, simultaneously, homeotype control reaction tubes were prepared. After pre-cooling and washing with PBS, the cells were counted by FACSCalibur flow cytometry (BD Biosciences). The results were analyzed by CellQuest software, with CD3^+^CD8^−^IL-17^+^ representing Th17 cells.

PBMC suspensions without stimulation were supplemented with human FITC-labeled anti-human CD4, APC-labeled anti-human CD25, and PE-labeled anti-human CD127, and incubated in the dark at room temperature for 15 minutes. Cells were washed with PBS twice, and then fixed with 300 μL PBS. Cells were detected by flow cytometry (FACSCalibur, BD Biosciences) and analyzed by CellQuest software, with CD4^+^CD25^high+^CD127^low^ representing Treg cells^43^.

### H&E, PAS, and IHC staining

The procedures were performed according to the kit manufacturer instructions. For H&E staining to detect inflammatory cells, the number of eosinophils, neutrophils, and lymphocytes were averaged from five different areas on each slide were evaluated microscopically at 400× magnification. IHC staining was scored by the same researcher under the same microscope, with yellow or brown staining considered positive signals. Mouse lung tissues were stained with p-PI3K, p-Akt, p-mTOR, and p-p70S6k antibodies and yellow/brown staining was scored under the microscope. Images were obtained at 200 × magnification, five positive areas per slice were selected for analysis, and optical density values were measured. The intensity of H&E, IHC, and PAS staining was evaluated semi-quantitatively using the following categories: 0 (no staining), 1+ (weak but detectable staining), 2+ (moderate or distinct staining), and 3+ (intense staining). For each specimen, an HSCORE value was derived by calculating the sum of the percentages of cells that were stained in each intensity category and multiplying that value by the weighted intensity of the staining, using the formula HSCORE = *Pi* (*i* + 1), where *i* represents the intensity score, and *Pi* is the corresponding percentage of cells^44^. In each slide, five different areas and 100 cells per area were evaluated microscopically with α*40 objective magnification. The percentage of cells at each intensity within these areas was determined at different times by two investigators blinded to the source of the samples, and the average of their scores was used.

### Western blot

Lung tissue from each mouse was sampled three times and prepared for western blot. The lung tissues were lysed in protein lysis buffer, and proteins were extracted and quantified by Coomassie blue staining. Proteins were detected by western blot using antibodies against p-PI3K (Cell Signaling Technology), p-Akt (Cell Signaling Technology), p-mTOR (Santa Cruz Biotechnology), and p-p70S6k (Epitomics). Proteins were quantified using Odyssey software 3.0 and normalized against β-actin as an internal control.

### Statistical analysis

All data were analyzed with SPSS v21.0 and were presented as mean ± standard deviation (SD). Each set of data was determined to conform to a normal distribution, analyzed by F-test for homogeneity of variance, and then subjected to a univariate analysis between groups in a multi-application, pairwise comparison with Bonferroni correction. Correlations were determined by Pearson correlation, with α = 0.05 set as the criterion for statistical significance.

## Electronic supplementary material

Supplementary information
