# Supplementary material for: Activation of the mTOR signaling pathway is required for asthma onset
Source: Sci Rep. 2017 Jul 3;7:4532. doi: 10.1038/s41598-017-04826-y (PMC5495772; doi:10.1038/s41598-017-04826-y)
Supplement: Supplementary file 1 — Supplementary information [file 41598_2017_4826_MOESM1_ESM.doc]

**­Activation of the mTOR signaling pathway is required for asthma onset**

Yanli Zhang^1*^, Ying Jing^2^, Junying Qiao^1^, Bin Luan^1^, Xiufang Wang^1^, Li Wang^1^, Zhe Song^1^

^1^Department of Pediatrics, the Third Affiliated Hospital of Zhengzhou University, Zhengzhou, Henan 450052, China

^2^School of Medicine for Basic Research, Zhengzhou University, Zhengzhou, Henan 450001, China

Correspondence to: yanlizhang2012@gmail.com

**Supplemental materials**

**Supplemental Table S1** Serum mTOR, Th17, Treg and cytokines from asthma patients who reached remission without treatment and with treatments.

| **Group** | **Remission w/ treatments** | | **Remission w/o treatments** | | **Statistics** | |
| --- | --- | --- | --- | --- | --- | --- |
| **n** | 19 | | 16 | |
|  | **mean** | **sd** | **mean** | **sd** | **F value** | **p-value** |
| **mTOR (pg/mL)** | 29.26 | 12.37 | 37.56 | 13.45 | 0.043 | p>0.05 |
| **IL-17 (pg/mL)** | 123.45 | 32.06 | 134.19 | 35.27 | 0.024 | p>0.05 |
| **TGF-β (pg/mL)** | 33.56 | 10.29 | 25.06 | 11.23 | 0.078 | p>0.05 |
| **IL-10 (pg/mL)** | 25.02 | 6.96 | 16.15 | 4.34 | 0.029 | p>0.05 |
| **Th17 (%)** | 5.12 | 2.11 | 2.98 | 1.81 | 0.121 | p>0.05 |
| **Treg (%)** | 5.18 | 1.09 | 7.45 | 1.45 | 0.078 | p>0.05 |
| **IL-4 (pg/mL)** | 16.25 | 3.12 | 25.37 | 4.23 | 0.357 | p>0.05 |
| **IFN-γ (pg/mL)** | 16.45 | 4.25 | 24.57 | 7.23 | 1.365 | p>0.05 |

**Table S1** Comparison of serum mTOR pathway and key measurements for Th17 and Treg between the asthma patients in the remission group who reached remission without treatment and with treatments of steroid budenoside and β2-adrenergic receptor agonists Terbutaline Sulphate Solution for Nebulization. mTOR, IL-17, TGF-β, IL-10, IL-4, and IFN-γ were measured by ELISA, whereas Th17 and Treg were quantified by flow cytometry. There were no statistical significances between these two groups. All data were analyzed with SPSS v21.0 and were presented as mean ± standard deviation (sd). Each set of data was determined to conform to a normal distribution, analyzed by F-test for homogeneity of variance, and then subjected to a univariate analysis between groups in a multi-application, pairwise comparison with Bonferroni correction.
